# Supplementary material for: Updating the Mechanism of Bicarbonate (HCO3−) Activation of Soluble Adenylyl Cyclase (sAC)
Source: Int J Mol Sci. 2025 Jul 3;26(13):6401. doi: 10.3390/ijms26136401 (PMC12250328; doi:10.3390/ijms26136401)
Supplement: Supplementary file 1 [file ijms-26-06401-s001.zip › ijms-3714352-supplementary.pdf]

International Journal of *Molecular Sciences*

MDPI

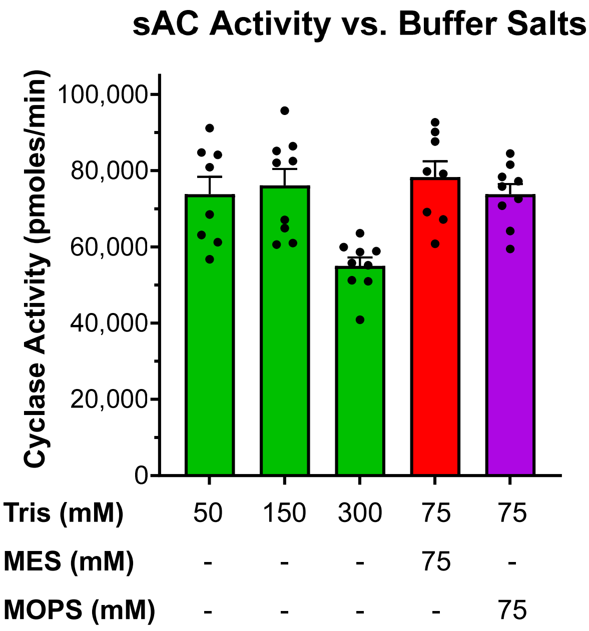

Supplemental Figure S1. Effects of buffer salts on sAC cyclase activity. *In vitro* cyclase activity assay of recombinant sAC_t in the presence of 10 mM of Mn^{+2}, 2 mM of ATP, and indicated concentrations of respective buffers. All conditions were adjusted to pH 7.5 prior to measuring cyclase activity. Mn^{+2} was used in this experiment for full catalytic activity. 300 mM of Tris decreased cyclase activity, either from excess Tris salt or counterion; therefore, subsequent experiments did not use higher than 150 mM of Tris. Bars represent standard errors of the means of triplicate determinations of experiments repeated three times; values were normalized to activity in the presence of 50 mM of Tris (pH 7.5).
